# Endoscope-assisted supraorbital eyebrow approach for the treatment of ethmoidal dural arteriovenous fistula

**DOI:** 10.3171/2025.7.FOCVID2580

**Published:** 2025-10-01

**Authors:** Marcello D’Andrea, Roberta Costanzo, Valentina Ricci, Dalila Fuschillo, Jacopo Visani, Luigino Tosatto

**Affiliations:** 1 Department of Neurosurgery, "Maurizio Bufalini" Hospital, Cesena, Italy; and; 2 Department of Neurosurgery, "Ospedali Riuniti Villa Maria-Cervello" Hospital, Palermo, Italy

**Keywords:** DAVF, eyebrow, endoscopic

## Abstract

Ethmoidal DAVFs represent 10%–15% of all intracranial DAVFs. They are generally associated with a higher bleeding risk due to more malignant drainage characteristics and, regardless of a patient’s symptoms, their treatment is highly recommended. Surgical disconnection has long been regarded as the gold-standard treatment for ethmoidal DAVFs, but several reports of successful endovascular treatments have been made.

The authors present a case of a 65-year-old patient with an occasional finding of ethmoidal DAVF treated through an endoscope-assisted supraorbital eyebrow approach. In this video, they explain the radiographical, anatomical, and surgical considerations and demonstrate the surgical technique.

The video can be found here: https://stream.cadmore.media/r10.3171/2025.7.FOCVID2580

## Transcript

This 2D video shows the use of the endoscope-assisted supraorbital eyebrow approach for the treatment of ethmoidal dural arteriovenous fistula.

### 0:35 Case Presentation.

We describe the case of a 65-year-old female with medical history positive for smoking and previous cervix squamous cell carcinoma.

The patient was on oncological follow-up and a PET scan was positive for a right nasopharyngeal hypermetabolism. The ENT evaluation was suspect for a vascular anomaly and the MRI showed a vascular malformation in the ethmoidal region.

The patient executed then a cerebral angiography, which showed an ethmoidal dural arteriovenous fistula fed from the ethmoidal arteries from both sides and unilateral venous drainage into a cortical vein. Both sides of the internal carotid artery are shown.

### 1:32 Current Treatment Options and Rationale for the Eyebrow Approach.

The current treatment options include: the microsurgical disconnection of the fistula, which is historically associated with higher obliteration rates and still is considered the gold-standard, and the endovascular treatment, either via the transarterial route through the ophthalmic artery or the transvenous route. These procedures are associated with higher procedural risks and lower obliteration rates and may require further interventions for definitive treatment.^1^

The current open approaches include the bifrontal craniotomy^2^ in cases of bilateral venous drainage of the fistula, and the pterional approach in cases of unilateral venous drainage of the fistula.^3^

The rationale for the supraorbital eyebrow approach^4^ is that is an approach that is currently used in our department for small/medium tumors of the anterior cranial fossa, it permits easy and direct access to the ethmoidal region, the surgical disconnection of ethmoidal dural AV fistulas is straightforward in cases with unilateral venous drainage, and it implies a smaller incision and craniotomy and so a shorter hospital stay.

### 2:59 Surgical Procedure.

The patient is positioned in the supine position with the head elevated 10° and rotated 30° on the opposite side.

The skin incision is made along the eyebrow without cutting the hair of the eyebrow, as previous studies have showed no increased risk of infection, and leaving the eyebrow intact allows for a better cosmetic result.

It is important that the incision is made laterally to the supraorbital notch to preserve the supraorbital nerve function. Care is taken to preserve the fibers of the orbicular oculi for reconstruction and better postoperative cosmetic results. Once the pericranium is exposed, it’s incised 1.5 cm superior to the supraorbital ridge and dissected away toward the temporalis muscle.

A small burr hole is done in the most anterior part of superior temporal line, made with a drill at the most anterior part of the superior temporal line, and two cuts are done, one with the standard craniotome and one with the Sonopet bone scalpel. The dura is opened in semicircular fashion and a small part of the frontal lobe is exposed. The opticocarotid cistern is opened to gain sufficient brain relaxation

Once the olfactory nerve comes into view it is followed anteriorly to expose the venous part of the dural AV fistula. With a slight change in microscope inclination the fistula is better visualized and all the cortical veins are exposed. At this point the QEVO inspection tool is brought into the field and the draining vein piercing the dura is exposed.

The dissection goes further and the vein is freed away. The venous varix is exposed and followed all the way to the cranial base where it enters.

A first ICG run is done to define the angioarchitecture of the fistula. Then, a temporary clip is placed to the point more proximal to the cranial base.

A second ICG shows initial slowing of the vein, then the culprit vein is coagulated and cut and the endoscope shows "darkening" of the venous component of the fistula, with normal times of circulation. The last ICG run shows complete disconnection of the fistula.

Bone is fixated with miniplates and reconstructed as usual.

### 7:02 Postoperative Clinical and Radiological Result.

The postoperative angiography shows complete exclusion of the AV fistula from both sides.

The patient was discharged on day 3 and had no neurological deficits. As you can see, this is the control at 1 month with some slight stupor of the frontal branch of the facial nerve.

### 7:40 Final Remarks.

The benefits of endoscope-assisted supraorbital eyebrow approach for the treatment of ethmoidal dural arteriovenous fistula is that it is a minimally invasive approach, with the limitation for the surgeon to work in small spaces; it has optical cosmetic results even if the surgeon has to work in narrow corridors and the surgeon has to use higher light intensity and that is correlated with lighting issues.

But the use of the endoscope overcomes all the issues related to the minimally invasive approaches, overcoming the lighting issue and guaranteeing a clearer view with an extended view angle.^5^

## Disclosures

The authors report no conflict of interest concerning the materials or methods used in this study or the findings specified in this publication.

## Author Contributions

Primary surgeon: D’Andrea. Assistant surgeon: Ricci, Fuschillo. Editing and drafting the video and abstract: D’Andrea, Costanzo, Visani. Critically revising the work: D’Andrea. Approved the final version of the work on behalf of all authors: D’Andrea. Supervision: Ricci, Tosatto.

## Supplemental Information

### Patient Informed Consent

The necessary patient informed consent was obtained in this study.

### Previous Presentations

Portions of this video were included in an oral presentation at the National Congress of the Italian Society of Neurosurgery (SINCH) held in Padua, Italy, October 12–14, 2023.

## References

[1] Berke CN Naik A Majmundar N Microsurgical versus endovascular treatment of ethmoidal dural arteriovenous fistulas: systematic review and meta-analysis with a single-center case series Neurosurg Focus 2024 56 3 E15 10.3171/2023.12.FOCUS23801 38428011

[2] Uchida M Tanikawa M Nishikawa Y Yamanaka T Ueki T Mase M Endoscope-controlled high frontal approach for dural arteriovenous fistula in anterior cranial fossa World Neurosurg 2023 175 e421 e427 37019304 10.1016/j.wneu.2023.03.116

[3] Gross BA Moon K Kalani MYS Clinical and anatomic insights from a series of ethmoidal dural arteriovenous fistulas at Barrow Neurological Institute World Neurosurg 2016 93 94 99 27241099 10.1016/j.wneu.2016.05.052

[4] Reisch R Perneczky A Ten-year experience with the supraorbital subfrontal approach through an eyebrow skin incision Neurosurgery 2005 57 4 Suppl 242 255 16234671 10.1227/01.neu.0000178353.42777.2c

[5] Arnaout MM Luzzi S Galzio R Aziz K Supraorbital keyhole approach: pure endoscopic and endoscope-assisted perspective Clin Neurol Neurosurg 2020 189 105623 31805490 10.1016/j.clineuro.2019.105623

